# An integrated transcriptomic and metabolomic atlas reveals the temporal regulation of benzylisoquinoline alkaloid biosynthesis and transport in developing opium poppy capsules

**DOI:** 10.3389/fpls.2026.1754793

**Published:** 2026-02-04

**Authors:** Qian Wang, Xiaofang Qie, Yun Zhen, Ruoshi Li, Zhaoyu Liu, Yanjun Zhang, Shilin Chen, Chi Song

**Affiliations:** 1 School of Pharmacy, Chengdu University of Traditional Chinese Medicine, Chengdu, China; 2 Institute of Herbgenomics, Chengdu University of Traditional Chinese Medicine, Chengdu, China; 3 College of Tropical Crops and College of Forestry, Hainan University, Haikou, China; 4 Wuhan Botanical Garden, Chinese Academy of Sciences, Wuhan, China

**Keywords:** benzylisoquinoline alkaloids (BIAs), capsule development, metabolomics, multi-omics analysis, opium poppy, transcriptomics, transporters

## Abstract

**Introduction:**

The opium poppy (*Papaver somniferum* L.) is the primary source of medically important benzylisoquinoline alkaloids (BIAs), including morphine and codeine. Nevertheless, the transcriptional regulatory networks and transport processes underlying the spatiotemporal accumulation of BIAs during capsule development remain incompletely understood.

**Methods:**

We performed an integrated transcriptomic and metabolomic analysis across five defined capsule developmental stages (S1–S5). Transcript–metabolite relationships were examined using global correlation analysis, trend analysis, and weighted gene co-expression network analysis (WGCNA). To identify putative BIA transporters, we applied a multi-tiered bioinformatic screening pipeline that combined hub-gene prioritization, transmembrane domain prediction, and functional annotation, followed by qRT-PCR validation of shortlisted candidates.

**Results:**

Metabolomic profiling revealed a clear developmental trajectory of BIA accumulation, with S4 emerging as a critical stage for BIA biosynthesis. Terminal alkaloids reached maximal levels at S4 and declined sharply at S5, suggesting the initiation of active transport and/or metabolic conversion at late development. Strong transcript–metabolite concordance enabled robust multi-omics integration. WGCNA identified 32 co-expression modules, among which the steelblue, brown, blue, and white modules showed the strongest associations with BIA metabolites, including the key intermediate thebaine. The integrated screening strategy and qRT-PCR validation ultimately highlighted *PsMATE1* and *PsEXS1* as the highest-confidence transporter candidates, supported by their multiple predicted transmembrane domains, membership in established transporter families, and expression patterns tightly synchronized with late-stage BIA accumulation.

**Conclusion:**

This study provides a comprehensive multi-omics resource and a systematic framework for transporter discovery in P. somniferum capsules. The identification of *PsMATE1* and *PsEXS1* offers prioritized targets for future functional characterization and advances understanding of the mechanisms controlling BIA transport and accumulation during capsule development.

## 1 Introduction

The opium poppy (*Papaver somniferum* L.) is an indispensable medicinal plant, serving as the primary commercial source for critical narcotic analgesics like morphine and codeine, as well as the precursor thebaine for semi-synthetic opioids such as oxycodone and naloxone (Vadhel et al., 2023; Májer and Németh, 2024). This unique economic and medicinal value stems from the plant’s sophisticated capacity to biosynthesize and accumulate over 100 distinct benzylisoquinoline alkaloids (BIAs), a large and structurally diverse class of nitrogen-containing specialized metabolites (Aghaali and Naghavi, 2024; Leibetseder et al., 2025). Beyond the well-known morphinan analgesics, the BIA family encompasses compounds with a broad spectrum of pharmacological activities, such as the antitussive noscapine, the vasodilator papaverine, and antimicrobial compounds such as sanguinarine, highlighting their significant potential for drug discovery and development (Hamilton and BAskett, 2000; Mahmoudian and Rahimi-Moghaddam, 2009; Beaudoin and Facchini, 2014).

The chemical synthesis of many complex BIAs, particularly (-)-morphine—a pentacyclic molecule with five chiral centers—remains economically non-viable due to its stereochemical complexity, cementing our reliance on plant-based production (Galanie et al., 2015). Consequently, a deep understanding of the biosynthetic machinery in opium poppy is crucial. The foundational BIA pathway begins with the condensation of two L-tyrosine-derived molecules, catalyzed by the pivotal enzyme norcoclaurine synthase (*NCS*) (Samanani et al., 2004; Lee and Facchini, 2011). This central intermediate is then progressively elaborated through a series of methylations, hydroxylations, and other modifications by enzymes such as O-methyltransferases and the cytochrome P450-dependent STORR protein, which is responsible for a critical stereochemical inversion that gates entry into the morphinan branch (Choi et al., 2002; Winzer et al., 2015). These enzymatic steps partition metabolic flux into distinct channels, ultimately leading to the production of the major alkaloids found in the plant’s latex (Facchini et al., 2007; Hagel and Facchini, 2013).

A defining feature of BIA metabolism in opium poppy is its extensive compartmentalization across multiple spatial scales. At the organ level, the network of laticifers embedded within the capsule and stem is the primary site for the biosynthesis, storage, and accumulation of high levels of BIAs, underscoring a strong organ specificity (Rezaei et al., 2018). At the cellular and subcellular levels, the pathway is remarkably partitioned between different cell types. A combination of *in situ* hybridization, proteomics, and laser microdissection studies has revealed that the early upstream enzymes (e.g., *NCS*, *6OMT*, *CNMT*) are predominantly localized to the sieve elements of the phloem, often associated with parietal smooth endoplasmic reticulum, while the late-stage enzymes responsible for the final steps of morphinan (e.g., *T6ODM*, *COR*) and noscapine biosynthesis are highly enriched in the laticifers (Bird et al., 2003; Onoyovwe et al., 2013; Ozber and Facchini, 2022). This spatial division of labor implies an obligatory intercellular transport of pathway intermediates from their site of initial synthesis in the phloem to their final site of modification and storage in the laticifers (Dastmalchi et al., 2019a).

This necessary movement of alkaloids and their precursors across multiple membranes and between distinct cell types positions transport processes as a major bottleneck and a critical, yet poorly understood, regulatory node in BIA flux. To date, research on BIA transporters has identified a few key players. Members of the *PUP/BUP* family, such as certain *BUP*s, have been functionally characterized as plasma membrane-localized transporters that facilitate the uptake of various BIAs into laticifers, suggesting a potential apoplastic route for alkaloid partitioning (Dastmalchi et al., 2019a). Other transporter families, including the Multidrug and Toxic Compound Extrusion (MATE) and ATP-binding cassette (ABC) transporters, are known in other plant systems to energize the vacuolar sequestration or plasma membrane efflux of alkaloids, making them strong candidates for similar roles in opium poppy (Takanashi, 2017). However, a systematic, genome-wide effort to identify, prioritize, and validate the full complement of transporters involved in BIA trafficking is conspicuously absent from the literature.

Furthermore, BIA accumulation is a dynamic process that unfolds over the course of capsule development. Transcriptomic and metabolomic studies have shown that the levels of key alkaloids, such as morphine, peak at capsule maturity, and that these shifts are accompanied by stage-specific expression of biosynthetic genes (Huang and Kutchan, 2000; Rezaei et al., 2018; Wang et al., 2025). While these studies provide snapshots of the process, a comprehensive, multi-omics integration that captures the continuous, co-varying trajectories of transcripts and metabolites throughout development is needed to reconstruct the underlying regulatory networks. In particular, the manner in which transporter gene expression is temporally coupled to the accumulation of specific BIA metabolites remains largely unexplored.

In this study, we hypothesize that the dynamic accumulation of BIAs during capsule development is orchestrated by co-varying gene expression trajectories, with transporters serving as critical bridges linking these patterns. To test this, we generated a comprehensive transcriptome and metabolome atlas across five developmental stages (S1–S5) and employed an integrated multi-omics approach. This strategy enabled us to identify key co-expression trends and modules strongly associated with BIA traits, particularly thebaine accumulation. From these networks, we prioritized a set of high-confidence transporter candidates based on their strong correlations, concordant trends, and central positions within key modules. Our findings provide a ranked, experimentally supported candidate list to guide future functional studies, thereby advancing a systems-level understanding of the temporal regulation governing BIA partitioning during capsule maturation.

## 2 Methods

### 2.1 Plant materials and sampling

**Figure 1:**
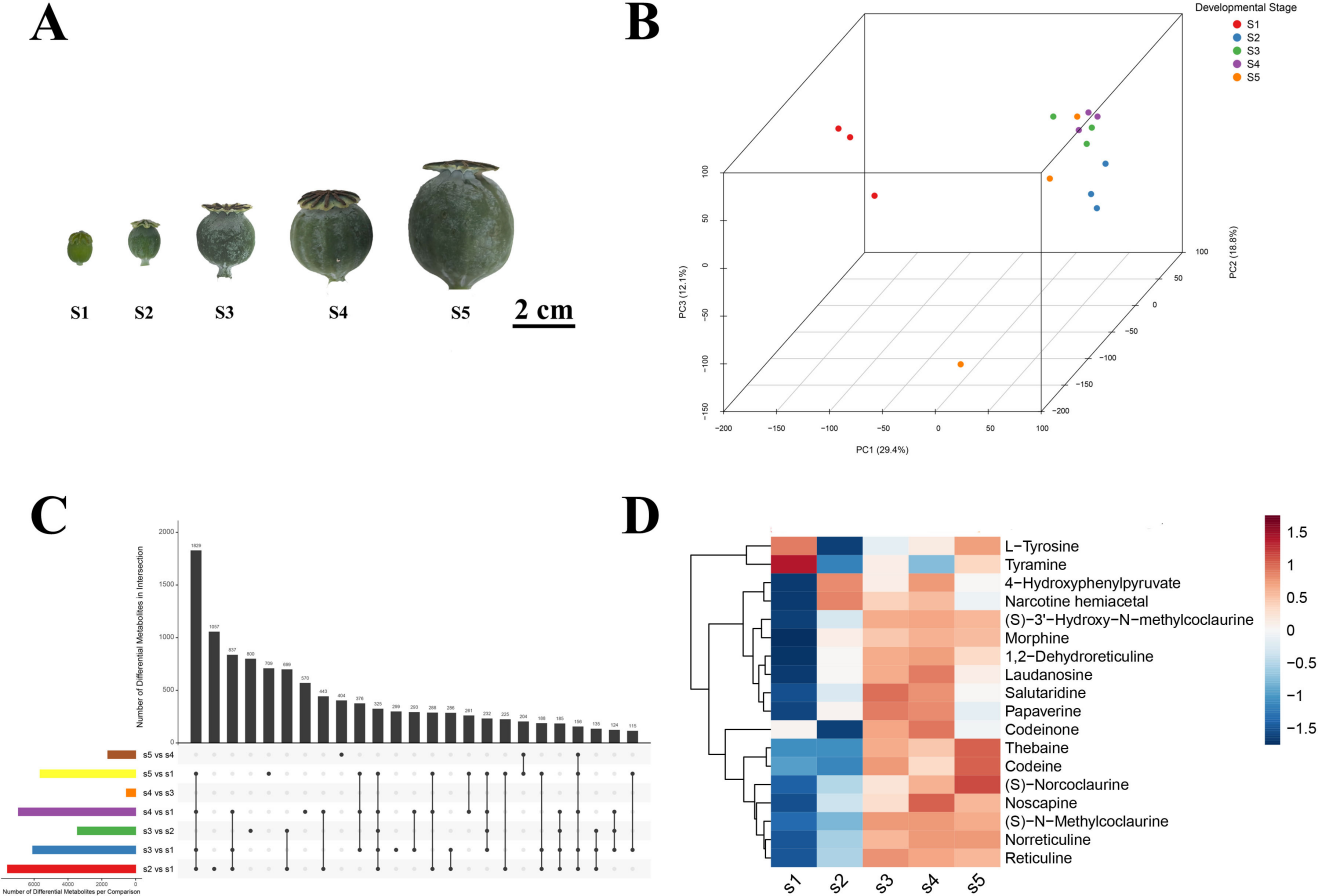
Morphological and metabolomic profiling of opium poppy capsules across five developmental stages. **(A)** Representative morphological images of opium poppy capsules at stages S1 to S5, defined by diameter and key developmental characteristics (see Methods for detailed criteria). Scale bar, 2 cm. **(B)** Three-dimensional Principal Component Analysis (3D PCA) of the metabolome across all five developmental stages (S1-S5). Data points are colored by developmental stage, illustrating the metabolic trajectory and clear separation of biological replicates (n=3 per stage) across the three principal components. **(C)** Upset plot summarizing the unique and shared differential metabolites across multiple pairwise comparisons between developmental stages. The bar chart on the left indicates the total number of differential metabolites identified per comparison, while the top bar chart and connected dots illustrate the size of specific intersections among these comparisons. **(D)** Heatmap depicting the accumulation patterns of benzylisoquinoline alkaloid (BIA)-related metabolites across the five developmental stages. The z-score normalized intensities are shown, with hierarchical clustering revealing co-accumulation patterns. The color scale from blue to red indicates low to high relative abundance.

Fresh capsules of opium poppy (*Papaver somniferum* L.) were collected from cultivated plots at Wuhan Botanical Garden (Wuhan, China) during late April under comparable environmental conditions to minimize diurnal and microclimatic variation. Sampling targeted five capsule developmental stages (S1–S5) defined by diameter and diagnostic morphology: S1 (< 1.00 cm) with non-lignified pericarps and persistent floral organs; S2 (1.25 ± 0.25 cm) marking the onset of cuticular wax deposition; S3 (2.25 ± 0.25 cm) showing evident vascular bundle differentiation; S4 (2.75 ± 0.25 cm) with the formation of apical dehiscence zones; and S5 (> 3.00 cm) characterized by fully lignified pericarps. Stage-specific morphological criteria and representative images are provided in **Figure 1A**. For each stage, at least three independent biological replicates were harvested from distinct plants following a randomized block scheme to balance potential batch effects. Immediately after excision, capsules were snap-frozen in liquid nitrogen, transported on dry ice, and stored at −80°C until RNA and metabolite extraction. All samples were processed following identical handling procedures to ensure comparability across stages.

### 2.2 Metabolite extraction and LC–MS analysis

Pericarps from five developmental stages (S1–S5; n = 3 per stage) were extracted with pre−chilled methanol:water (7:3, v/v) spiked with isotope−labeled internal standards (d3−Leucine, 13C9−Phenylalanine, d5−Tryptophan, 13C3−Progesterone). Approximately 50 mg tissue was homogenized by bead−beating, sonicated on ice (30 min), precipitated at −20°C (1 h), and centrifuged (16,000 g, 15 min, 4°C). Supernatants were 0.22 μm−filtered; pooled quality−control (QC) samples were prepared by combining equal aliquots from all extracts.

Chromatography used a C18 column (2.1 × 100 mm, 1.9 μm) with water (0.1% formic acid) and acetonitrile (0.1% formic acid) at 0.3 mL/min and 40°C. The gradient ramped 5–95% B over 22 min with wash/re−equilibration to 30 min. Data were acquired on a Q Exactive HF in ESI+ and ESI− modes: full−scan m/z 125–1,500 (ESI+) and 100–1,500 (ESI−) at 120,000 resolutions, followed by data−dependent MS/MS.

Raw files were processed in Compound Discoverer v3.3 for peak detection, alignment, blank subtraction, and feature grouping (precursor error <5 ppm, fragment error <10 ppm, RT tolerance <0.2 min). Features present in blanks at ≥20% of the mean sample intensity were removed. Peak areas were normalized to internal standards and corrected for signal drift using QC−based LOESS. Putative annotations were assigned via exact mass, isotope fitting, and MS/MS spectral matching against BMDB, mzCloud, and ChemSpider.

### 2.3 RNA extraction, library preparation, and RNA sequencing

Capsule pericarps from the five developmental stages (S1–S5) were collected as described above, with three independent biological replicates per stage. Total RNA was isolated using the RNeasy Plant Mini Kit (Qiagen, 74904) with on-column DNase I treatment (Qiagen, 79254) to remove residual genomic DNA. RNA integrity was assessed using an Agilent 2100 Bioanalyzer; only samples with suitable RIN and A260/280 were advanced. RNA quantity was measured by Qubit fluorometry (Thermo Fisher Scientific). Poly(A)+ libraries were prepared with the MGIEasy RNA Library Prep Kit (MGI Tech, 1000006384) following the manufacturer’s protocol, using ∼1 µg total RNA per sample. Library insert sizes were 250–300 bp as determined by Bioanalyzer. Pooled libraries were sequenced on the DNBSEQ platform to generate 40 million 150-bp paired-end reads per sample.

Raw reads were evaluated with FastQC (v0.11.9). Adapter trimming and quality filtering were performed using fastp (v0.21.0). Clean reads were aligned to the *Papaver somniferum* reference genome (NCBI BioProject PRJNA435796) using STAR (v2.7.9a) with splice-aware settings. Differential expression was analyzed with DESeq2 (v1.34.0) using a design matrix including developmental stages; significance was set at |log2FoldChange| ≥ 1 and p values < 0.1. Functional enrichment of DEGs was conducted with clusterProfiler (v4.2.2) for Gene Ontology and KEGG terms using a genome-appropriate background.

### 2.4 Global correlation between transcriptome and metabolome

To assess the overall association between transcriptional and metabolic dynamics throughout capsule development, a Pearson correlation analysis was performed between all quantified transcripts and metabolites. The correlation matrix was constructed using the normalized and transformed expression (VST) and abundance (log2-scaled) data across all biological replicates (n=3) of the five developmental stages (S1-S5). The strength of the linear relationship for each transcript-metabolite pair was evaluated by calculating Pearson’s correlation coefficient (r).

### 2.5 Time-course trend analysis

Temporal patterns across the five developmental stages were analyzed separately for transcriptomic and metabolomic profiles using fuzzy c-means clustering implemented in the Mfuzz R package (v2.58.0). For transcriptomic data, differentially expressed genes were identified by comparing each subsequent stage (S2-S5) against the S1 baseline using thresholds of |log2FoldChange| ≥ 2 and p-value ≤ 0.05. For metabolomic data, differential metabolites were screened using thresholds of | log2FoldChange| ≥ 0.5 and p-value ≤ 0.1 relative to the S1 baseline.

The resulting matrices of stage-averaged values (FPKM for transcripts, normalized intensities for metabolites) were standardized by z-score transformation and clustered into eight temporal patterns using identical fuzzy c-means parameters. Functional enrichment analysis was performed cluster-specifically: transcript clusters underwent KEGG pathway enrichment analysis using a custom hypergeometric test implementation, while metabolite clusters were subjected to metabolic pathway enrichment analysis based on Sub Pathway annotations.

### 2.6 Weighted gene co-expression network analysis

Gene co-expression networks were constructed using the WGCNA Shiny application in TBtoolsII (Chen et al., 2023; Wang et al., 2024). Genes with FPKM > 1 in at least one developmental stage were retained for analysis. A soft-thresholding power of 14 was selected based on scale-free topology fit (R² > 0.8) and mean connectivity (< 500) criteria using signed network topology. The adjacency matrix was transformed to a topological overlap matrix (TOM), and module identification was performed with dynamic tree cutting (minimum module size = 30; deepSplit = 2), followed by module merging at a height cut of 0.15.

Module eigengenes (MEs) were correlated with developmental stages (S1-S5, encoded ordinally) and with 18 BIA-related metabolite abundances (M1-M18, see **Supplementary Table 1**). Associations were quantified using biweight midcorrelation with Benjamini-Hochberg FDR correction (FDR < 0.05). Within modules significantly associated with BIAs or developmental stages, hub genes were identified based on intramodular connectivity (kME) and gene significance metrics.

For network visualization, module subnetworks were extracted using TOM-based edge weights from significantly trait-associated modules. Networks were imported into Cytoscape (v3.10.4), where candidate hub genes were prioritized by combined network centrality measures (degree, betweenness) and biological context (known BIA pathway membership).

### 2.7 Functional domain and subcellular localization prediction

The protein sequences of candidate transporter genes identified from WGCNA were analyzed using DeepTMHMM (version 1.0.24) to predict transmembrane helices and subcellular localization. Sequences were submitted to the public web server (https://dtu.biolib.com/DeepTMHMM) using default parameters.

To identify conserved functional domains within the candidate proteins, sequences were queried against the Pfam (version 36.0) and Conserved Domain Database (CDD, version 3.20) using the online batch search tools provided by the NCBI (https://www.ncbi.nlm.nih.gov/Structure/cdd/wrpsb.cgi) and the Pfam website (https://www.ebi.ac.uk/interpro/search/sequence/).

### 2.8 Quantitative real-time PCR validation

Total RNA was isolated from pericarp tissues across five developmental stages (S1–S5; n = 3 biological replicates per stage) to validate the expression patterns of candidate transporter genes identified through transcriptome-metabolome integration. Gene-specific primers for qRT-PCR were designed using NCBI Primer-BLAST (https://www.ncbi.nlm.nih.gov/tools/primer-blast/) and synthesized by Sangon Biotech (Shanghai, China), sequences provided in **Supplementary Table 2**. Following the manufacturer’s protocol for the RevertAid Reverse Transcription Premix (Thermo Scientific, M16325), RNA samples were treated with DNase I (37°C for 5 min) and inactivated with EDTA (65°C for 10 min). First-strand cDNA was synthesized in a 20 µL reaction at 42°C for 30 min, with reaction termination at 95°C for 5 min.

qRT-PCR was performed using the ChamQ Universal SYBR qPCR Master Mix (Vazyme, Q713) on a JLM-QX series real-time PCR system. Each 10 µL reaction contained gene-specific primers and was run under the following conditions: initial denaturation at 95°C for 30 s, followed by 40 cycles of 95°C for 10 s and 60°C for 30 s. Amplification specificity was confirmed by melt curve analysis. All assays included three technical replicates, alongside no-template and no-reverse transcription controls. Relative gene expression was calculated using the 2^−ΔΔCt^ method, with *Actin* as the internal reference. Data analysis and visualization were performed using R (version 4.2.1).

### 2.9 Data analysis and statistics

Unless otherwise specified in the preceding sections, all data analysis and statistical computations were performed within the R programming environment (version 4.2.1). Data visualization was generated using ggplot2 and other R packages. For multiple hypothesis testing, p-value adjustment was conducted using the Benjamini-Hochberg false discovery rate (FDR) procedure where applicable. A significance threshold of FDR < 0.05 or p-value < 0.05 was adopted unless stated otherwise.

## 3 Results

### 3.1 Metabolomic landscapes define five developmental stages of opium poppy capsules

To delineate the dynamic progression of capsule development, opium poppy fruits were systematically categorized into five distinct stages (S1 to S5) based on diameter and definitive morphological markers (**Figure 1A**). The S1 stage was characterized by persistent floral organs and a non-lignified, wax-free pericarp. A key morphological transition was observed at S2, marked by the conspicuous deposition of a white waxy coating that persisted throughout all subsequent stages.

Global metabolomic profiling via Principal Component Analysis (PCA) revealed a clear and progressive separation across the five developmental stages (**Figure 1B**), thereby validating our staging system. This separation was further supported by the distinct clustering patterns observed among biological replicates and between experimental groups (**Supplementary Figure 1**). The metabolomes of S1, S2, and S5 were highly distinct from all other stages. Notably, S1 and S5 occupied the most distant positions in the PCA space, underscoring a profound shift in chemical composition between the initiation and termination of development. While S3 and S4 were also separable from other stages, their close clustering suggested a period of metabolic continuity, potentially reflecting their sequential progression through a shared phase of rapid secondary metabolism.

We next quantified stage-specific metabolic shifts by identifying differentially accumulated metabolites (DAMs) in all pairwise comparisons between consecutive stages (**Figure 1C**), using a threshold of |log2(fold change)| ≥ 1 and p-value ≤ 0.1. The most substantial metabolic reprogramming occurred during the S1-to-S2 transition, with 4,325 metabolites significantly upregulated and 3,249 downregulated. This dramatic reshuffling coincides with key morphological events, such as the initiation of wax deposition, and likely represents a broad metabolic switch from primary growth to active secondary metabolism. A similarly extensive reshuffling was observed between S1 and S4. In stark contrast, the S3-to-S4 transition involved the fewest DAMs (282 upregulated and 264 downregulated), reinforcing their metabolic similarity as indicated by PCA.

We next focused on the spatiotemporal dynamics of benzylisoquinoline alkaloids (BIAs). The early precursors, L-tyrosine and tyramine, were most abundant in S1 (**Figure 1D**), consistent with their role in providing carbon and nitrogen skeletons for the rapid initiation of BIA synthesis. The levels of intermediate BIAs, including thebaine and codeine, generally increased throughout development, with a notable transient dip at S4 before rebounding at S5, suggesting a potential for dynamic interconversion or regulated transport during the late stages. A pivotal finding was the accumulation pattern of the terminal BIA products, morphine, papaverine, and noscapine, which peaked sharply at S4 followed by a marked decline at S5. This decline is consistent with the onset of active degradation, conversion, or transport processes at the final stage of capsule maturation. The subsequent decline provides evidence consistent with the onset of active transport, reallocation, or conversion processes late in development. Collectively, these stage-specific accumulation profiles establish the five developmental stages as critical checkpoints orchestrating BIA biosynthesis, interconversion, and storage.

### 3.2 Stage-specific transcriptional reprogramming underpins capsule development

**Figure 2:**
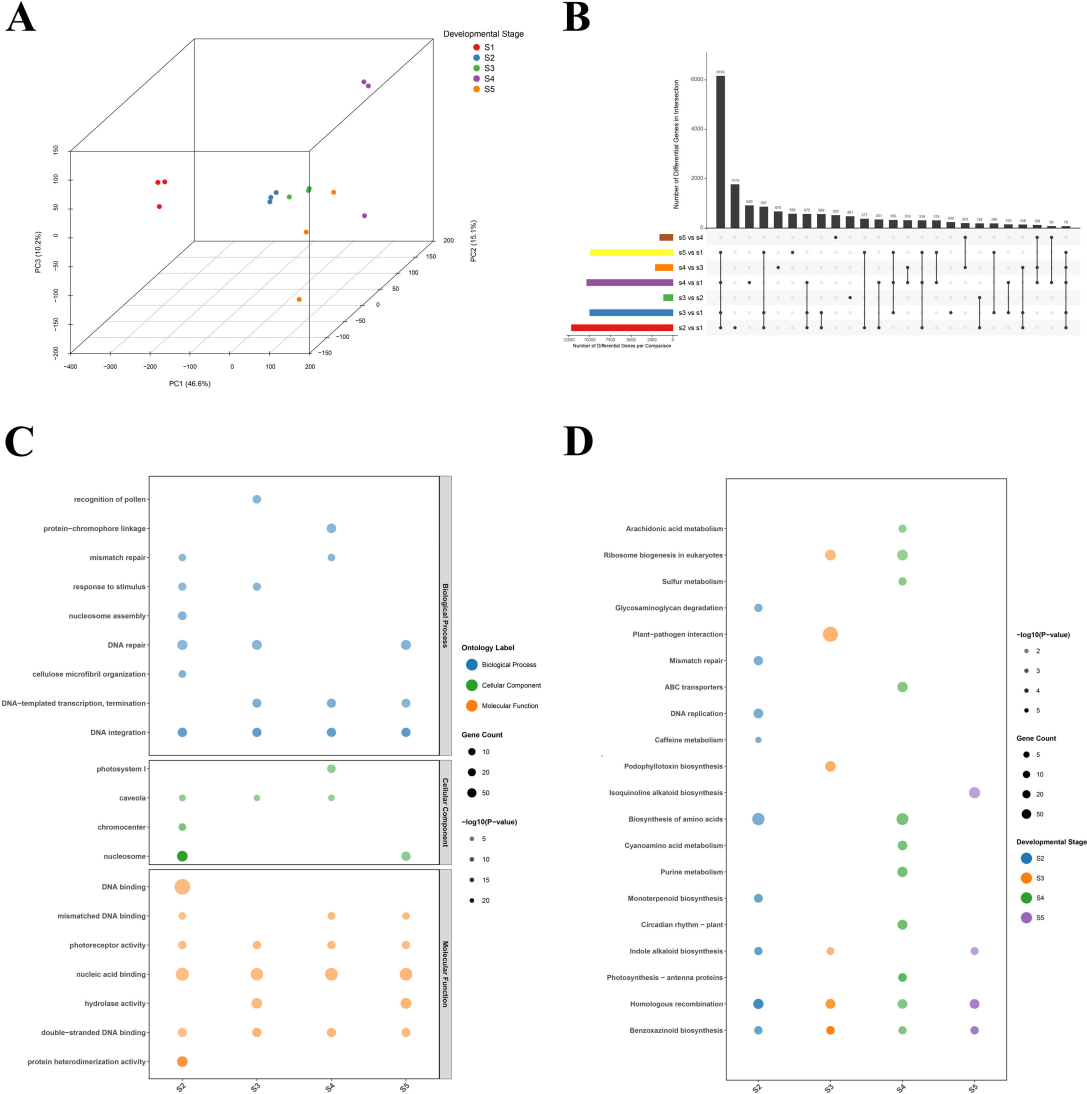
Transcriptomic dynamics and functional enrichment during opium poppy capsule development. **(A)** Three-dimensional Principal Component Analysis (3D PCA) of the transcriptome across the five developmental stages (S1-S5). Data points are colored by developmental stage, illustrating the transcriptional trajectory and clear separation of biological replicates (n=3 per stage) across the three principal components. **(B)** Upset plot visualizing the unique and shared differentially expressed genes (DEGs) from pairwise comparisons between consecutive developmental stages. The bar chart on the left shows the total number of DEGs identified in each comparison, while the top bar chart and connected dots highlight the complexity of gene expression changes, with many DEGs being specific to particular stage transitions. **(C)** Bubble heatmap of Gene Ontology (GO) enrichment analysis for DEGs across stages. Selected significantly enriched terms (FDR < 0.05) are displayed. The bubble color represents the GO ontology: Biological Process, Cellular Component, and Molecular Function. The bubble size corresponds to the number of DEGs mapped to each term (gene count), and the color intensity (within each ontology) represents the enrichment significance (-log10(P-value)). **(D)** Bubble heatmap of Kyoto Encyclopedia of Genes and Genomes (KEGG) pathway enrichment analysis for DEGs. The plot shows the top significantly enriched metabolic and biosynthetic pathways (FDR < 0.05). The bubble color represents the developmental stage (S2, S3, S4, S5) in which the enrichment was identified. The bubble size corresponds to the gene count, and the color intensity (within each stage) represents the enrichment significance (-log10(P-value)).

Principal component analysis (PCA) of the transcriptome data revealed a clear global shift in gene expression across capsule development (**Figure 2A**). This global separation was reinforced by the distinct clustering patterns observed among biological replicates and between experimental groups in **Supplementary Figure 2**. The S1 stage exhibited the greatest separation from all subsequent stages, indicating a unique transcriptional state during initial capsule formation. The distinct clustering pattern of the transcriptome, particularly the closer proximity of S3 and S5 in the PCA plot compared to the metabolome, suggests a more complex relationship between mRNA abundance and metabolite accumulation.

An Upset plot visualized the complex sets of differentially expressed genes (DEGs) identified from all pairwise stage comparisons, using a threshold of |log2(fold change)| ≥ 0.5 and a P-value < 0.1 (**Figure 2B**). Consistent with the PCA, comparisons involving the S1 stage yielded the highest number of DEGs. The S1 versus S2 comparison revealed 6,925 upregulated and 5,250 downregulated genes, highlighting this initial transition as a period of profound transcriptional reprogramming. The continuity of this transcriptional program was further evidenced by the S4 *vs* S3 comparison, which yielded a notably small set of DEGs and no uniquely enriched functional categories in our analysis. This transcriptional stability between S3 and S4 stands in marked contrast to the metabolic shifts observed in this window, reinforcing the hypothesis that post-transcriptional and transport-level regulation predominates during this critical phase.

Gene Ontology (GO) and Kyoto Encyclopedia of Genes and Genomes (KEGG) enrichment analyses were streamlined to focus on terms directly pertinent to alkaloid biology, transcriptional regulation, and transport (**Figures 2C, D**; **Supplementary Tables 3**, **4**). This focused approach confirmed the sequential activation of specialized metabolism, with ‘isoquinoline alkaloid biosynthetic process’ enriched in S3 *vs* S2, and terms like ‘alkaloid metabolic process’ and ‘xenobiotic detoxification by transmembrane export’ enriched in S5 *vs* S4. KEGG analysis specifically highlighted the exclusive enrichment of ‘Isoquinoline alkaloid biosynthesis’ at S5, correlating with terminal BIA accumulation.

**Figure 3:**
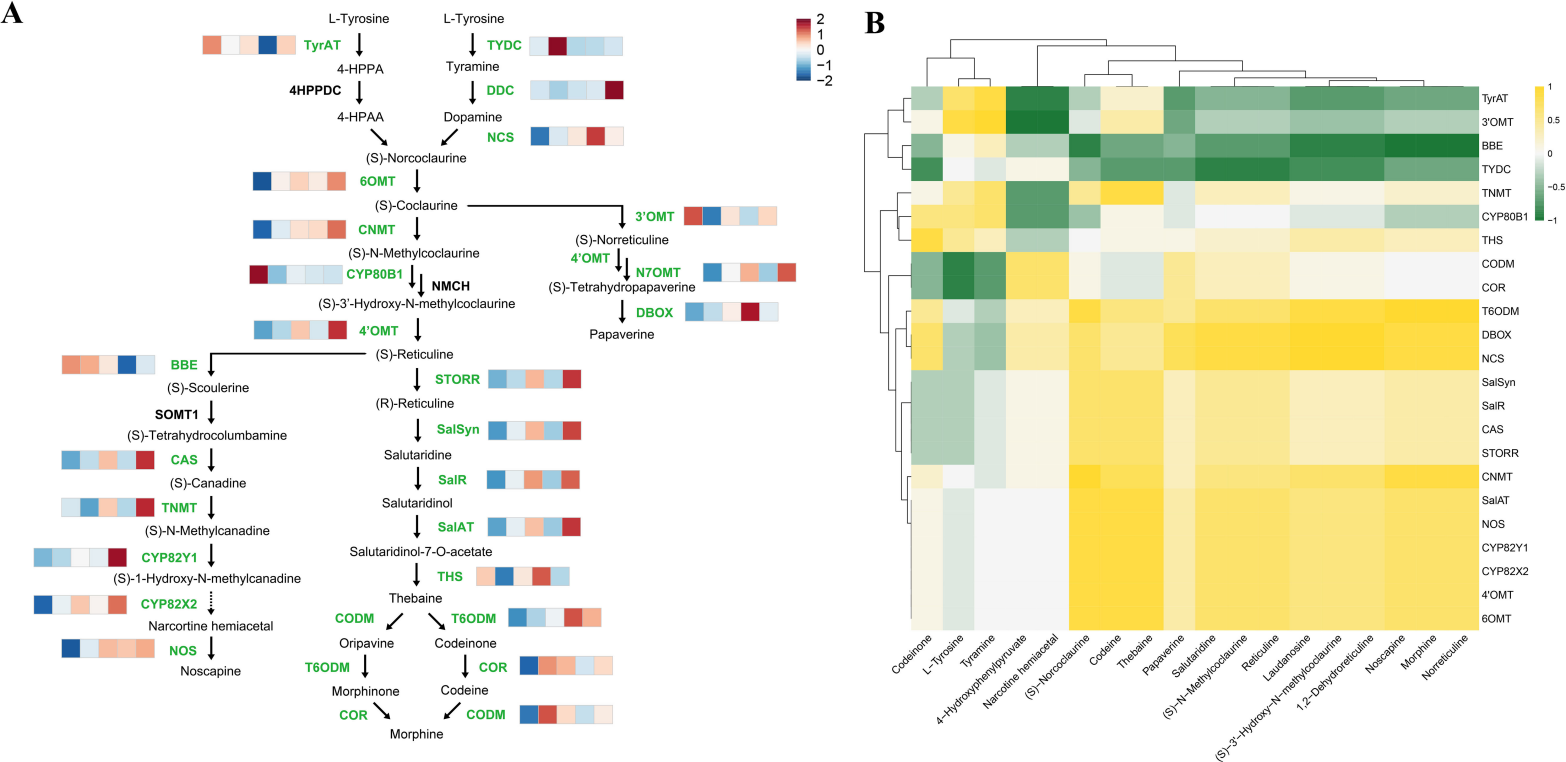
Dynamic expression and correlation of BIA biosynthetic genes across capsule development. **(A)** Heatmap of BIA biosynthetic gene expression across five developmental stages (S1 to S5). The heatmap depicts the expression levels of 23 known BIA biosynthetic genes, arranged according to their positions in the canonical pathway. Each row represents one gene, and each column represents a developmental stage (left to right: S1 to S5). Gene names highlighted in green in the pathway diagram indicate those successfully detected in our transcriptome data. Major branches for noscapine, morphine, and papaverine biosynthesis are indicated. Expression values are Z-score normalized per gene and visualized using a red-blue color scale, with red indicating high expression and blue indicating low expression. **(B)** Correlation heatmap between BIA biosynthetic genes and metabolites. Spearman correlation coefficients were calculated between the expression levels of the 23 BIA biosynthetic genes (rows) and the accumulation levels of 18 quantified BIA metabolites (columns) across all samples from stages S1 to S5. The color scale from green to yellow indicates the correlation strength, with yellow representing strong positive correlation and green representing weak or negative correlation. Only gene-metabolite pairs with a nominal significance of p < 0.05 are displayed.

To systematically anchor our analysis within the established biochemical framework, we mapped the expression dynamics of 23 known BIA biosynthetic genes across the five developmental stages (**Figure 3A**). This detailed view of the canonical pathway revealed a clear transcriptional logic underlying BIA production. Upstream genes, such as *TyrAT* and *TYDC*, were highly expressed during the early stages (S1/S2). In contrast, genes associated with late pathway branches, including *CAS* and *TNMT* for noscapine, *SalR* and *SalAT* for morphine, and *N7OMT* and *DBOX* for papaverine, showed peak expression at later stages. A particularly striking pattern was observed within the morphine branch: genes from *STORR* to *SalAT* displayed an expression pattern that increased from S1 to S3, sharply declined at S4, and partially recovered at S5. Conversely, genes responsible for the final conversion to thebaine, namely *THS* and *T6ODM*, exhibited their highest expression precisely at S4. This inverse expression pattern between mid-pathway and terminal thebaine-synthesizing enzymes, coupled with the previously noted peak and subsequent decline of thebaine itself at S4, strongly implicates this stage as a pivotal node for orchestrating BIA flux, potentially through active transport of this key intermediate.

To further investigate the coordination between transcript and metabolite levels within the BIA pathway, we performed a correlation analysis between the 23 biosynthetic genes and 18 quantified BIA metabolites across all samples (**Figure 3B**). Spearman correlation analysis identified 13 gene-metabolite pairs as significantly correlated (FDR < 0.05), with an additional 65 pairs showing nominal significance (p < 0.05). Several key relationships emerged. Notably, *DBOX*, a downstream enzyme in the papaverine branch, showed significant positive correlations with laudanosine—a reported precursor to papaverine—as well as with other papaverine pathway intermediates ((S)-3’-Hydroxy-N-methylcoclaurine and 1,2-Dehydroreticuline). This pattern is consistent with *DBOX*’s position at a metabolic branch point. The overall limited number of strong, direct correlations likely reflects the functional complexity of enzymes like *T6ODM*, *COR*, and *CODM*, which operate in multiple morphine-pathway sub-branches, as well as the existence of unresolved steps in the noscapine and papaverine pathways.

### 3.3 Integrated multi-omics analysis reveals coordinated metabolic and transcriptional dynamics

**Figure 4:**
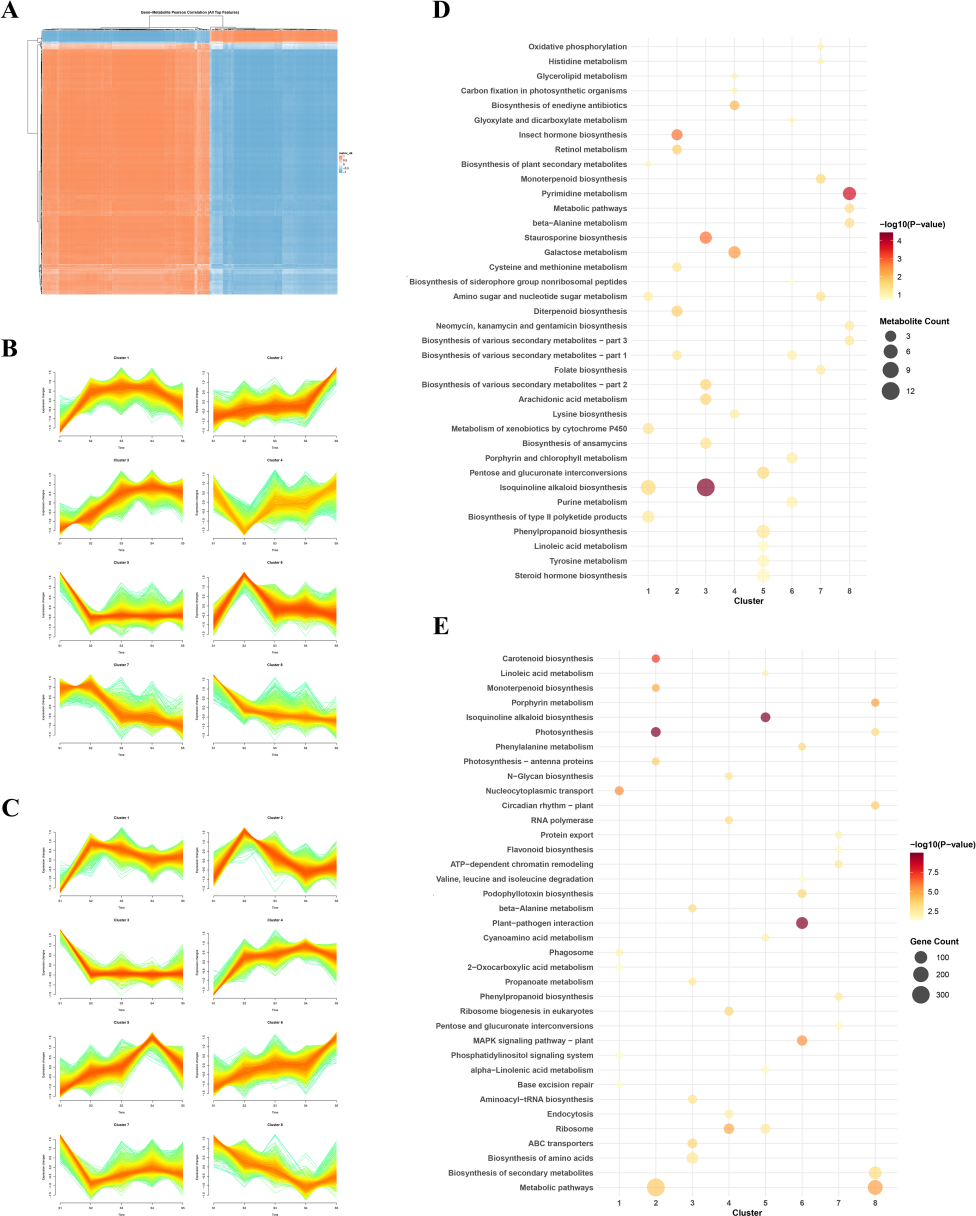
Integrated analysis of transcriptomic and metabolomic dynamics during capsule development. **(A)** Global Pearson correlation heatmap between all detected transcripts and metabolites across all samples. The color intensity represents the correlation coefficient (r), illustrating the overall coordination between transcriptional and metabolic networks. **(B)** Temporal trend analysis of metabolomic profiles. Metabolites were clustered into eight distinct patterns using fuzzy c-means clustering, depicting their accumulation trajectories from stages S1 to S5. **(C)** Temporal trend analysis of transcriptomic profiles. Differentially expressed genes were clustered into eight distinct patterns using fuzzy c-means clustering, depicting their accumulation trajectories from stages S1 to S5. **(D)** Metabolic pathway enrichment bubble plot for the metabolite clusters shown in **(B)**. Significantly enriched pathways (P-value < 0.05) are displayed for each cluster. Bubble color indicates the enrichment significance (-log10(P-value)), and size represents the number of metabolites in the pathway. **(E)** KEGG pathway enrichment bubble plot for the gene clusters shown in **(C)**. The top enriched pathways (selected by P-value) for each transcriptional trend are shown. Bubble color corresponds to the enrichment significance (-log10(P-value)), and size represents the gene count. This analysis links specific expression trends to key biological pathways.

To first assess the global relationship between transcriptional and metabolic changes during capsule development, we performed a Pearson correlation analysis between all expressed genes and accumulated metabolites across the five stages. The resulting correlation matrix revealed an extensive and robust positive association between the two datasets (**Figure 4A**). The strength of this coordination was underscored by a high mean absolute correlation coefficient of 0.88 and a median of 0.76. Strikingly, 92.06% of all variable pairs exhibited a strong correlation (|r| > 0.7; **Supplementary Table 5**). At the gene level, 80,212 genes (52.35% of all genes analyzed) were strongly positively correlated (r > 0.7) with one or more metabolites, while 60,847 genes (39.71%) showed strong negative correlations (r < -0.7). This indicates that shifts in gene expression are systematically linked to changes in metabolite abundance. This pervasive interconnectivity strongly suggests the existence of a tightly regulated network, prompting us to investigate specific co-expression and co-accumulation patterns.

We next employed Mfuzz trend analysis to delineate the dominant temporal patterns of metabolites and transcripts. Fuzzy clustering of the differential metabolites identified several distinct accumulation trends (**Figure 4B**). Among these, clusters 1, 2, and 3 displayed a consistent increase throughout development, aligning with the overarching process of capsule growth and maturation. Pathway enrichment analysis of these clusters revealed that both cluster 1 and cluster 3 were significantly enriched in the ‘Isoquinoline alkaloid biosynthesis’ pathway (**Supplementary Table 6**; **Figure 4C**). This finding not only confirms that key BIAs follow a progressive accumulation trajectory but also powerfully validates the initial patterns observed in the stage-resolved metabolite heatmap (**Figure 4D**).

A parallel trend analysis of the transcriptomic data uncovered analogous patterns for gene expression (**Figure 4D**). Specifically, clusters 4, 5, and 6 exhibited steadily increasing expression profiles from S1 to S4. KEGG enrichment analysis confirmed that cluster 5 was also significantly associated with ‘Isoquinoline alkaloid biosynthesis’ (**Supplementary Table 7**; **Figure 3E**). This concordance between the rising trends of BIA pathway genes and the accumulation of BIA metabolites provides compelling bidirectional evidence for a concerted biosynthetic program from S1 to S4. Intriguingly, Cluster 6 was significantly enriched for Plant–pathogen interaction, consistent with the well-established integration of specialized-metabolite pathways with defense signaling and trafficking. The subsequent decline in gene expression at S5, coupled with metabolic shifts, implies a developmental transition away from active biosynthesis towards storage and transport.

### 3.4 Weighted gene co-expression network analysis identifies BIA-associated modules and hub genes

#### 3.4.1 Network construction and identification of thebaine-associated modules

**Figure 5:**
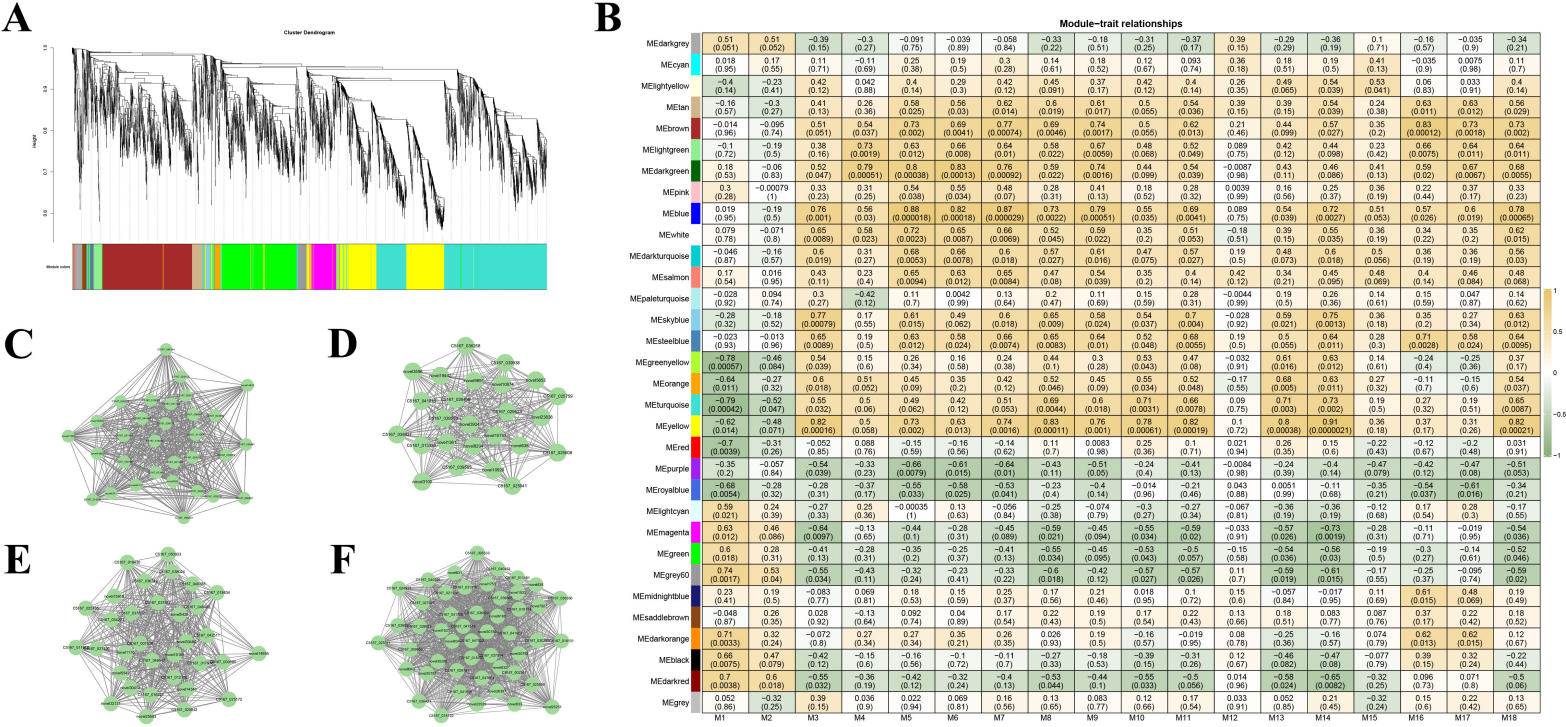
Weighted Gene Co-expression Network Analysis (WGCNA) identifies key modules associated with BIA accumulation. **(A)** Cluster dendrogram of genes based on their expression patterns across all developmental stages. Each leaf in the tree represents a single gene, and the major branches (distinguished by color) represent the co-expression modules identified by dynamic tree cutting. The height represents the dissimilarity based on topological overlap. **(B)** Heatmap depicting the correlation between module eigengenes and the abundance of 18 BIA-related metabolites (M1-M18, **Supplementary Table 1**). Each row represents a module, and each column represents a metabolite. The color intensity of each cell indicates the Pearson correlation coefficient (r), with asterisks denoting the statistical significance. Modules with significant associations to specific BIAs were selected for downstream analysis. **(C–F)** Cytoscape visualization of the gene co-expression networks for the key BIA-associated modules: **(C)** brown, **(D)** blue, **(E)** steelblue, and **(F)** white. In each subnetwork, nodes represent genes with high degree centrality, and edges represent the strongest topological overlap (TOM) connections between them.

To move beyond individual correlations and decipher the higher-order transcriptional programs coordinating capsule development, we constructed a weighted gene co-expression network. A scale-free topology was achieved with a soft-thresholding power of 14 (**Supplementary Figure 3**, Scale-free topology fit), and the analysis of ~8,000 highly expressed and variable genes partitioned the transcriptome into 32 distinct modules (**Figure 5A**), with sizes ranging from the large ‘turquoise’ (1,407 genes) and ‘blue’ (1,076 genes) modules to the small ‘paleturquoise’ module (32 genes).

We next interrogated the association between module eigengenes and the abundance of key BIA metabolites (**Figure 5B**). Ten modules demonstrated correlations (|r| > 0.5) with one or more BIA pathway metabolites (**Supplementary Table 8**). Among these, the ‘yellow’ module exhibited the highest average correlation coefficient (r = 0.633), while the ‘blue’ module was associated with the greatest number of distinct BIA metabolites (n = 15).

Given its pivotal role as a central biosynthetic intermediate and an established substrate for characterized BIA transporters (Dastmalchi et al., 2019a; Ozber et al., 2022; Ozber and Facchini, 2022), we focused our subsequent analysis on thebaine (M16). Two modules, ‘steelblue’ and ‘brown’, showed exceptionally strong positive correlations with thebaine accumulation (|r| > 0.7). Hub genes from the ‘brown’ module (834 genes) exhibited a progressive increase in expression from S1 to S5, whereas those from the smaller ‘steelblue’ module (28 genes) peaked sharply at S4 (**Supplementary Figures 4A, B**). To contextualize our findings within known BIA transport mechanisms, we queried our network using the sequence of BUP1, a known benzylisoquinoline uptake transporter (Dastmalchi et al., 2019a). Two homologous genes were identified, residing in the ‘blue’ (r = 0.57 with thebaine) and ‘white’ (r = 0.34) modules, respectively. The ‘blue’ module hub genes exhibited an S4-peaking expression pattern similar to the ‘steelblue’ module (**Supplementary Figure 4C**).

We then extracted the four most promising modules—’steelblue’, ‘brown’, ‘blue’, and ‘white’—for detailed subnetwork visualization in Cytoscape (**Figures 5C–F**). By applying module-specific topological thresholds, we distilled these networks to their core, highly interconnected hub genes. An integrated analysis of intramodular connectivity (kME), gene significance (GS) for thebaine, and network centrality was then employed to further narrow down the candidate list, yielding a refined set of high-priority targets for subsequent functional analysis.

#### 3.4.2 A multi-tiered bioinformatics pipeline prioritizes high-confidence transporter candidates

**Figure 6:**
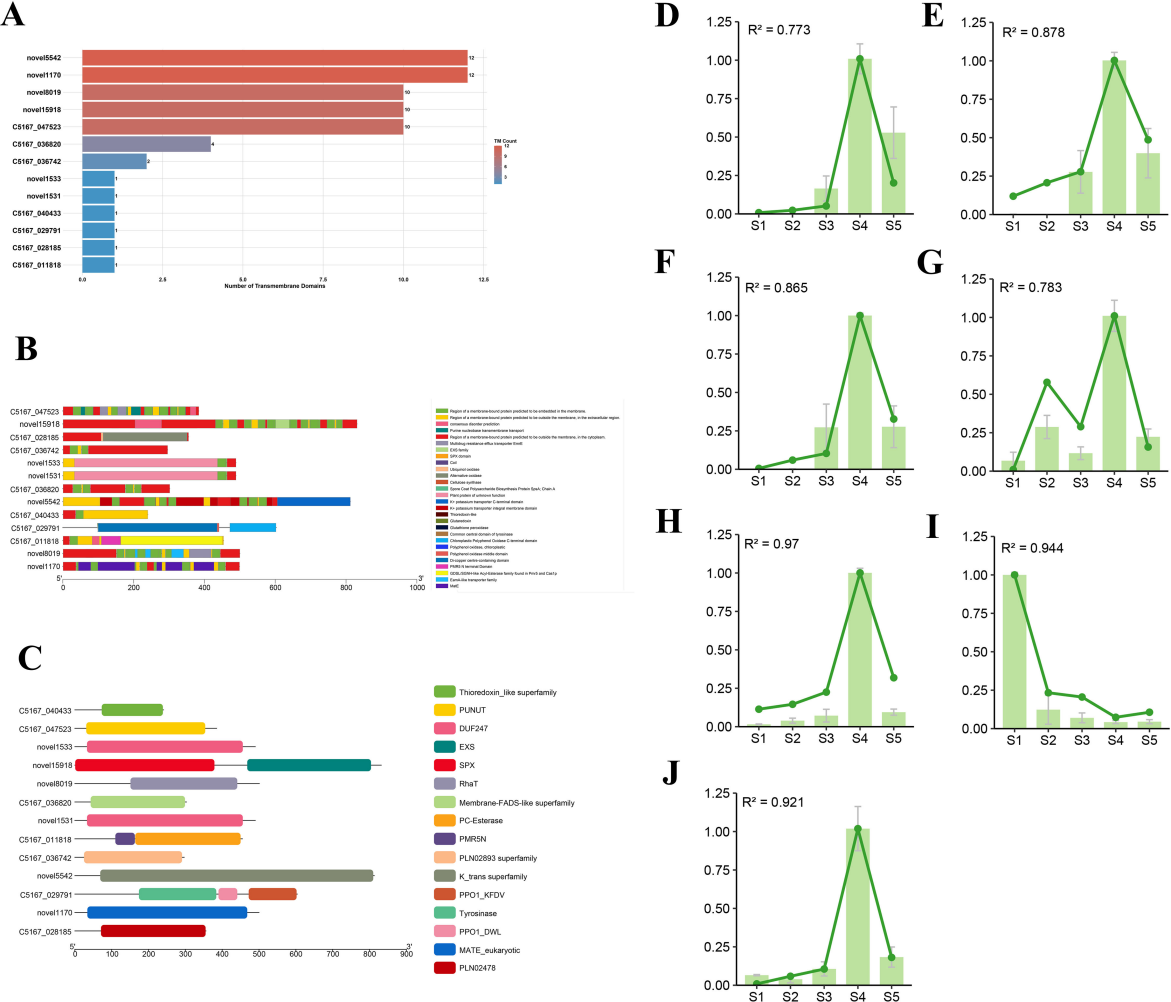
Domain characterization and expression validation of candidate transporter genes. **(A)** Transmembrane helix prediction for candidate transporter proteins using DeepTMHMM. The histogram displays the number of predicted transmembrane domains (TMDs) per protein, with proteins grouped and sorted by TMD count. **(B, C)** Protein domain architecture of candidate transporters as predicted by **(B)** Pfam and **(C)** Conserved Domain Database (CDD). Horizontal bars represent individual protein sequences, with colored blocks indicating the position and type of identified functional domains. **(D–J)** Validation of transcript abundance for seven candidate transporter genes by qRT-PCR. Expression levels of **(D)***PsBUP1* (C5167_047523), **(E)***PsEXS1* (novel15918), **(F)***PsFAD5* (C5167_036820), **(G)***PsHAK5* (novel5542), **(H)***PsMATE1* (novel1170), **(I)***PsRhaT1* (novel8019), and **(J)***PsTBR23* (C5167_011818) across five developmental stages (S1-S5) are shown. Bar plots (mean ± SD, n=3 biological replicates) represent qRT-PCR data, while dotted lines with points represent corresponding RNA-Seq data. The Pearson correlation coefficient (R²) between qRT-PCR and RNA-Seq data is indicated for each gene.

To systematically prioritize candidates capable of membrane transport, we employed a multi-tiered bioinformatic screening pipeline. DeepTMHMM analysis of the candidate genes from the four key modules identified 13 proteins possessing predicted transmembrane domains (TMDs). A graphical summary of the transmembrane topologies for these 13 candidates is presented in **Figure 6A**. Among the TMD-containing proteins, C5167_047523 was confirmed as the known BIA transporter *BUP1* (Dastmalchi et al., 2019a), which served as a positive control and was predicted to have 10 TMDs. Notably, several other candidates, including novel1170 (12 TMDs), novel5542 (12 TMDs), novel8019 (10 TMDs) and novel15918 (10 TMDs), were predicted to possess multiple TMDs, a structural hallmark of functional transporters, marking them as high-priority targets.

Pfam and Conserved Domain Database (CDD) analyses of these 12 top candidates, alongside *BUP1*, provided critical functional insights into their potential roles (**Figures 6B, C**). The Pfam analysis revealed 29 distinct domains. Crucially, several known transporter families were identified, most notably the MATE (Multidrug and Toxic Compound Extrusion) family, to which *PsMATE1* (novel1170) belongs, which is well-established in the vacuolar sequestration of plant alkaloids (Takanashi, 2017). Other prominent transporter-associated domains included MATE and Multidrug resistance efflux transporter EmrE. Additionally, domains linked to membrane systems or ion homeostasis, such as the K+ potassium transporter domain and SPX domain, were detected, suggesting proteins that may indirectly influence BIA transport by maintaining electrochemical gradients.

The CDD analysis corroborated and functionally refined these findings, identifying 16 protein families. Direct associations with transport were confirmed for the MATE eukaryotic family and expanded to include the PUNUT (nucleotide-sugar transporter), RhaT (rhamnose transporter), and EXS families. Furthermore, the presence of domains like the Membrane-FADS-like and K+ trans superfamily, involved in general membrane integrity and potassium transport, respectively, further supported the candidates’ potential roles in processes reliant on membrane potential, a key driver of secondary transport.

By integrating the results from Pfam and CDD domain analyses, with a focus on domains directly implicated in transport or membrane function, we further refined our candidate list to six high-priority transporter genes. These final candidates, assigned systematic names based on their predominant domain annotations, are *PsFAD5* (C5167_036820), *PsMATE1* (novel1170), *PsEXS1* (novel15918), *PsTBR23* (C5167_011818), *PsHAK5* (novel5542), and *PsRhaT1* (novel8019).

### 3.5 Validation of candidate transporter gene expression by qRT–PCR

To validate the RNA-seq expression patterns and refine our candidate list, we performed qRT-PCR analysis on the six prioritized genes alongside the known transporter PsBUP1. The qRT-PCR results demonstrated a high degree of consistency with the transcriptomic data, which was quantified by a significant positive correlation (p < 0.001) and high R² values (Pearson correlation coefficient squared) exceeding 0.7 across the tested genes.

The expression dynamics revealed distinct temporal patterns (**Figures 6D–J**). Crucially, four candidates—*PsEXS1*, *PsFAD5*, *PsMATE1*, and *PsTBR23*—exhibited an expression trend highly concordant with *BUP1*, characterized by a gradual increase from S1 to S3, a sharp peak at S4, and a subsequent decline at S5. This pattern aligns with the peak accumulation period for key BIA metabolites. In contrast, *PsHAK5* showed substantial expression at S2 in addition to its S4 peak, and *PsRhaT1* was predominantly expressed at S1, with minimal expression during later stages.

Integrating these expression profiles with our prior structural and functional annotations allowed for a final, robust prioritization. *PsMATE1* and *PsEXS1* emerged as the foremost candidates. Both possess multiple predicted transmembrane domains (12 and 10, respectively)—a key structural prerequisite for transporters. Furthermore, they are annotated to protein families with established roles in transport: *PsMATE1* belongs to the MATE eukaryotic family, renowned for the vacuolar sequestration of plant alkaloids, and PsEXS1 is associated with the EXS family, implicated in the transport of diverse substrates. Their co-expression with *BUP1* at S4, the critical phase for BIA flux, provides compelling *in vivo* evidence for their potential involvement in the same biological process. Therefore, we propose *PsMATE1* and *PsEXS1* as the highest-priority candidates for future functional characterization of BIA transport in opium poppy.

## 4 Discussion

This study constructs a dynamic transcriptomic and metabolomic atlas across five developmental stages of the opium poppy capsule, systematically delineating the temporal program governing benzylisoquinoline alkaloid (BIA) biosynthesis and accumulation. By integrating co-expression network analysis with a multi-tiered bioinformatic screening strategy, we have precisely identified two high-confidence candidate genes, *PsMATE1* and *PsEXS1*, implicated in BIA transport.

While poppy capsule morphology and BIA content have been studied, particularly across different cultivars and tissues (Desgagné-Penix et al., 2012; Wang et al., 2025), a time-resolved analysis of the developing capsule—the primary site of alkaloid harvest—has been lacking. Our study fills this critical gap, revealing that the physical expansion of the capsule is underpinned by profound internal metabolic reprogramming. The observed shift from high precursor (L-tyrosine, tyramine) abundance in S1 to peak end-product (morphine, papaverine) accumulation in S4 exemplifies the classic transition from primary growth to active specialized metabolism, a developmental switch well-documented in other plant systems (Erb and Kliebenstein, 2020; Maeda and Fernie, 2021). The subsequent decline in these final BIAs at S5, coupled with a transient dip and rebound in intermediates like thebaine and codeine, provides evidence consistent with the onset of active transport, reallocation, or conversion processes late in development. This metabolic pattern aligns with the established model of “late-stage synthesis and storage” where the final steps of BIA biosynthesis are enriched in laticifers (Ozber and Facchini, 2022), creating a demand for efficient metabolite trafficking.

The transcriptome data robustly corroborated these metabolic dynamics, revealing a stage-specific transcriptional underpinning for BIA accumulation. Integrating a pathway-anchored view of BIA metabolism with our developmental atlas provides a mechanistic framework for interpreting the S4 metabolic peak and the subsequent S5 decline. This spatiotemporal co-expression provides powerful *in vivo* evidence for their functional involvement in late-stage BIA metabolism indicate a developmental shift from early precursor provisioning, toward late-stage, branch-specific commitment, consistent with a broader growth-to-specialized-metabolism transition described across plant systems (Maeda and Fernie, 2021). Notably, the non-uniform and in some cases non-monotonic expression patterns within the morphinan branch around S4—including differential behavior among segments that include the key stereochemical “gatekeeper” step catalyzed by *STORR* (Winzer et al., 2015)—argue against a simple model in which end-product accumulation is driven solely by synchronized transcriptional upregulation of the entire linear pathway. Instead, when considered together with the gene–metabolite Spearman correlation heatmap, which shows only partial concordance between transcript abundance and metabolite pools, our data are most consistent with a multi-layered regulation in which pathway branching, multifunctional enzymes, and transport/compartmentation can decouple mRNA dynamics from steady-state metabolite levels (Ziegler et al., 2009; Shitan and Yazaki, 2013; Ozber and Facchini, 2022). This interpretation supports our working hypothesis that S4 represents a critical developmental window during which late-stage biosynthetic capacity and transport demand become tightly coordinated to enable efficient alkaloid allocation and storage.

Our subsequent WGCNA co-expression analysis, a powerful tool for dissecting complex metabolic pathways (Langfelder and Horvath, 2008; Pan et al., 2025), delineated 32 modules and identified 10 as strongly associated with BIA profiles. This “module-first” strategy is exceptionally efficient for gene discovery in specialized metabolism (Wisecaver et al., 2017). Among these, we prioritized four key modules as regulatory “hotspots,” which contained not only known biosynthetic genes like *THS* and *NISO*—enzymes critical for late-stage thebaine formation and codeinone synthesis, respectively (Chen et al., 2018; Dastmalchi et al., 2019b)—but also a pool of uncharacterized genes. The presence of the known thebaine transporter *BUP1* (Dastmalchi et al., 2019a) within this network further validated its biological relevance for BIA transport.

From these modules, we implemented a stringent bioinformatic pipeline to distinguish likely transporters from other hub genes. This integrated approach—combining network centrality, DeepTMHMM transmembrane prediction (Hallgren et al., 2022), and Pfam/CDD domain analysis (Wang et al., 2023; Paysan-Lafosse et al., 2025)—refined our list to six high-priority candidates. Final qRT-PCR validation revealed that *PsMATE1* and *PsEXS1* exhibited expression patterns tightly synchronized with both *BUP1* and the peak of BIA accumulation at S4. This spatiotemporal co-expression provides correlative *in vivo* evidence supporting their potential functional involvement in late-stage BIA metabolism.

The predicted protein structures and domains of *PsMATE1* and *PsEXS1* are consistent with a putative transporter function. Both possess multiple transmembrane domains, a structural hallmark of transporters. *PsMATE1* belongs to the MATE family, members of which are established mediators of vacuolar sequestration for various plant alkaloids via H^+^ antiport, as demonstrated by *CjMATE1* for berberine in *Coptis japonica* (Takanashi, 2017) and *NtJAT1/2* in *Nicotiana tabacum* (Morita et al., 2009). *PsEXS1*, containing EXS domains, is associated with export functions. While EXS-family proteins like PHO1 are best known for phosphate homeostasis (Secco et al., 2012; Wege et al., 2016), their core role in transmembrane export positions *PsEXS1* as a compelling candidate for the efflux of small molecules like BIAs (Bay and Turner, 2012; Vetal and Poirier, 2023).

Based on our findings, we propose a model wherein core co-expression modules, activated during S4, coordinately upregulate BIA biosynthetic machinery (*THS*, *NISO*) and specific transport systems (*PsMATE1*, *PsEXS1*) to facilitate the synthesis and subsequent directional transport of alkaloids into laticifers for storage.

We acknowledge that the evidence presented here, while strong, is correlative. The definitive functional characterization of *PsMATE1* and *PsEXS1* as BIA transporters requires future validation through heterologous expression and functional assays. Furthermore, applying spatial omics technologies will be crucial to resolve the precise cellular and subcellular localization of these transport processes, offering a next-resolution view of BIA compartmentalization.

## 5 Conclusion

In summary, our integrated multi-omics analysis of opium poppy capsule development delineates a clear temporal program for benzylisoquinoline alkaloid (BIA) metabolism, identifying the S4 stage as a critical biosynthetic and regulatory hub. By employing a systematic gene discovery pipeline that combined co-expression network analysis with transmembrane domain prediction and functional domain annotation, we successfully prioritized two high-confidence transporter candidates, *PsMATE1* and *PsEXS1*. Their structural features as multi-pass transmembrane proteins, coupled with expression patterns tightly synchronized to BIA accumulation, provide a strong rationale and a prioritized foundation for their future functional characterization, thereby advancing our understanding of BIA transport mechanisms.

## Data Availability

The transcriptomic data presented in this study are deposited in the NCBI Sequence Read Archive (SRA) repository, under BioProject accession number PRJNA1406540. Specifically, the samples analyzed in this paper correspond to BioSample accessions SAMN54773813 to SAMN54773827.
