# Supplementary material for: An integrated transcriptomic and metabolomic atlas reveals the temporal regulation of benzylisoquinoline alkaloid biosynthesis and transport in developing opium poppy capsules
Source: Front Plant Sci. 2026 Feb 4;17:1754793. doi: 10.3389/fpls.2026.1754793 (PMC12913367; doi:10.3389/fpls.2026.1754793)
Supplement: Supplementary file 1 [file DataSheet1.pdf]

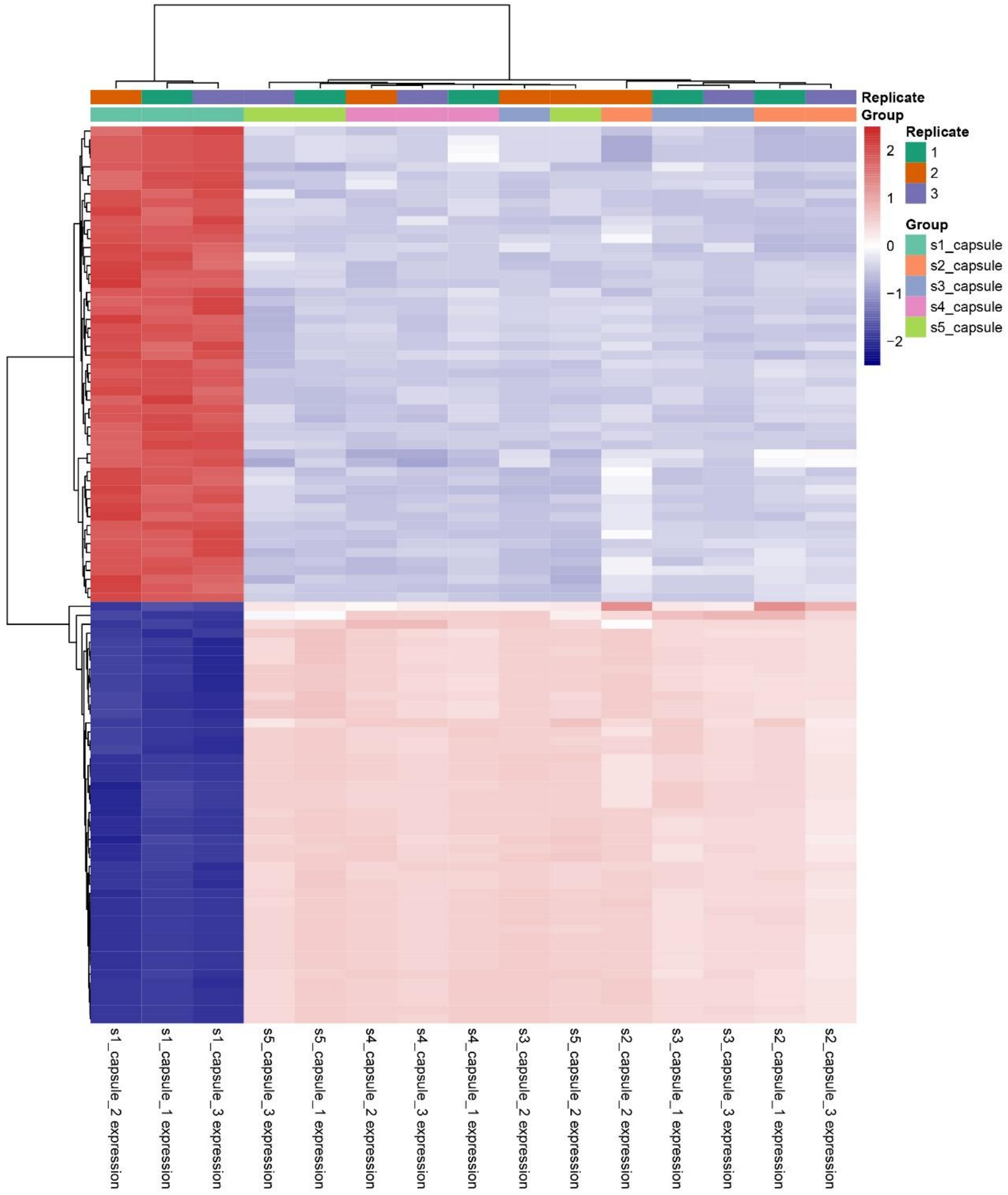


**Supplementary Figure 1. Hierarchical clustering heatmap of metabolomic profiles across capsule developmental stages.**

The heatmap displays the relative abundance (Z-score per metabolite) of the top 100 metabolites showing the strongest group-specific effect identified by differential analysis. The input data were log2-transformed values with an offset of 1 (log2p1). Samples from five distinct developmental stages (S1 to S5) are arranged by columns, with three biological replicates per stage (1-3). Metabolites are clustered by rows. Z-score normalization was

performed per metabolite across all samples. Hierarchical clustering was performed using Euclidean distance and complete linkage. The color scale from blue to red indicates low to high relative abundance, respectively.
