# Supplementary material for: An integrated transcriptomic and metabolomic atlas reveals the temporal regulation of benzylisoquinoline alkaloid biosynthesis and transport in developing opium poppy capsules
Source: Front Plant Sci. 2026 Feb 4;17:1754793. doi: 10.3389/fpls.2026.1754793 (PMC12913367; doi:10.3389/fpls.2026.1754793)
Supplement: Supplementary file 2 [file DataSheet2.pdf]

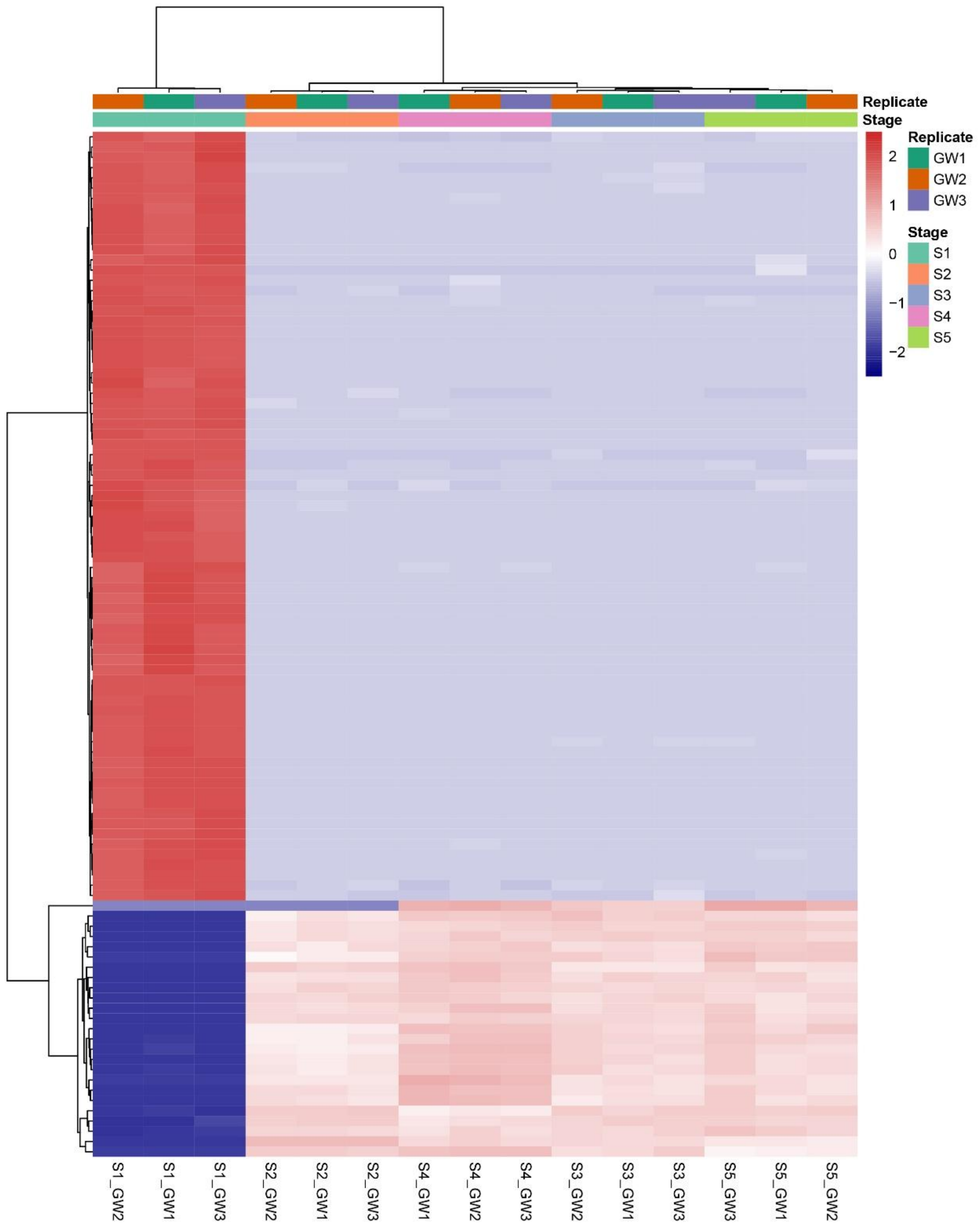

Supplementary Figure 2. Hierarchical clustering heatmap of transcriptomic profiles across capsule developmental stages.

The heatmap displays the relative expression (Z-score per gene) of the top 100 genes showing the strongest stage-specific effects identified by differential analysis. Samples from five distinct developmental stages (S1 to S5) are arranged by columns, with three biological replicates per stage (GW1-GW3). Genes are clustered by rows. Z-score normalization was performed per gene across all samples. Hierarchical clustering was performed using

Euclidean distance and complete linkage. The color scale from blue to red indicates low to high relative expression, respectively.
