# Supplementary material for: An integrated transcriptomic and metabolomic atlas reveals the temporal regulation of benzylisoquinoline alkaloid biosynthesis and transport in developing opium poppy capsules
Source: Front Plant Sci. 2026 Feb 4;17:1754793. doi: 10.3389/fpls.2026.1754793 (PMC12913367; doi:10.3389/fpls.2026.1754793)
Supplement: Supplementary file 3 [file DataSheet3.pdf]

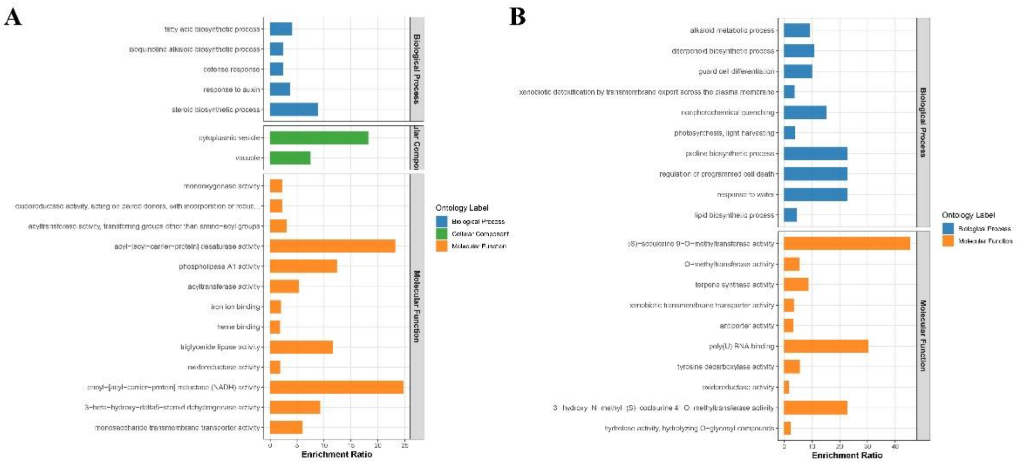

Supplementary Figure 3. Gene Ontology (GO) enrichment analysis of differentially expressed genes (DEGs) in key developmental transitions.

(A) Significantly enriched GO terms (FDR < 0.05) for DEGs identified in the S3 versus S2 comparison. (B) Significantly enriched GO terms (FDR < 0.05) for DEGs identified in the S5 versus S4 comparison. In both panels, the bar length corresponds to the gene count (number of DEGs) associated with each term. GO terms are categorized into Biological Process (BP), Cellular Component (CC), and Molecular Function (MF).
