# Supplementary material for: An integrated transcriptomic and metabolomic atlas reveals the temporal regulation of benzylisoquinoline alkaloid biosynthesis and transport in developing opium poppy capsules
Source: Front Plant Sci. 2026 Feb 4;17:1754793. doi: 10.3389/fpls.2026.1754793 (PMC12913367; doi:10.3389/fpls.2026.1754793)
Supplement: Supplementary file 4 [file DataSheet4.pdf]

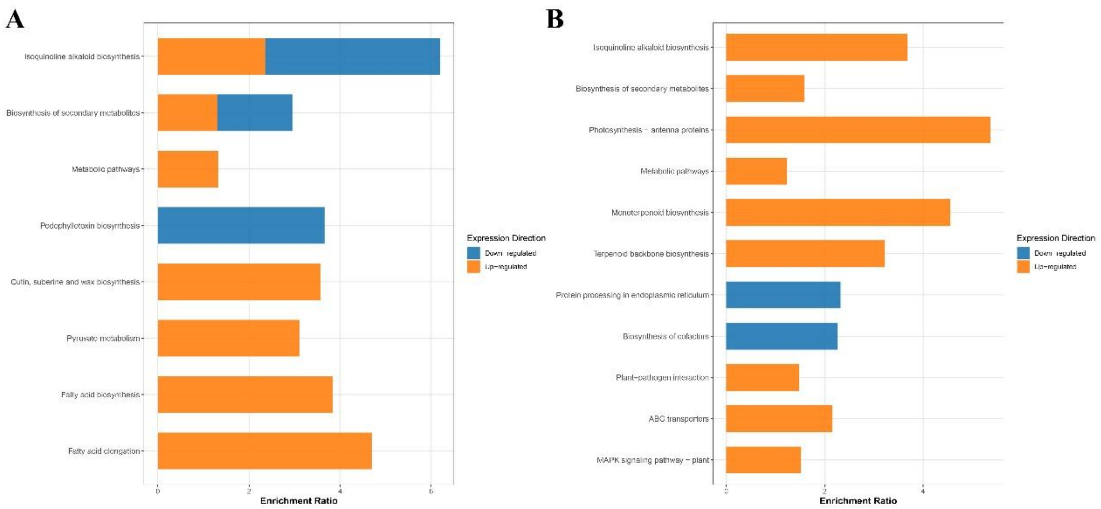

Supplementary Figure 4. Kyoto Encyclopedia of Genes and Genomes (KEGG) pathway enrichment analysis of differentially expressed genes (DEGs) in key developmental transitions.

(A) Top significantly enriched KEGG pathways (FDR < 0.05) for DEGs from the S3 versus S2 comparison. (B) Top significantly enriched KEGG pathways (FDR < 0.05) for DEGs from the S5 versus S4 comparison. In both panels, the bar length corresponds to the gene count, and the color indicates the direction of regulation (up- or down-regulated) for the genes contributing to the pathway enrichment.
