# Supplementary material for: An integrated transcriptomic and metabolomic atlas reveals the temporal regulation of benzylisoquinoline alkaloid biosynthesis and transport in developing opium poppy capsules
Source: Front Plant Sci. 2026 Feb 4;17:1754793. doi: 10.3389/fpls.2026.1754793 (PMC12913367; doi:10.3389/fpls.2026.1754793)
Supplement: Supplementary file 5 [file DataSheet5.pdf]

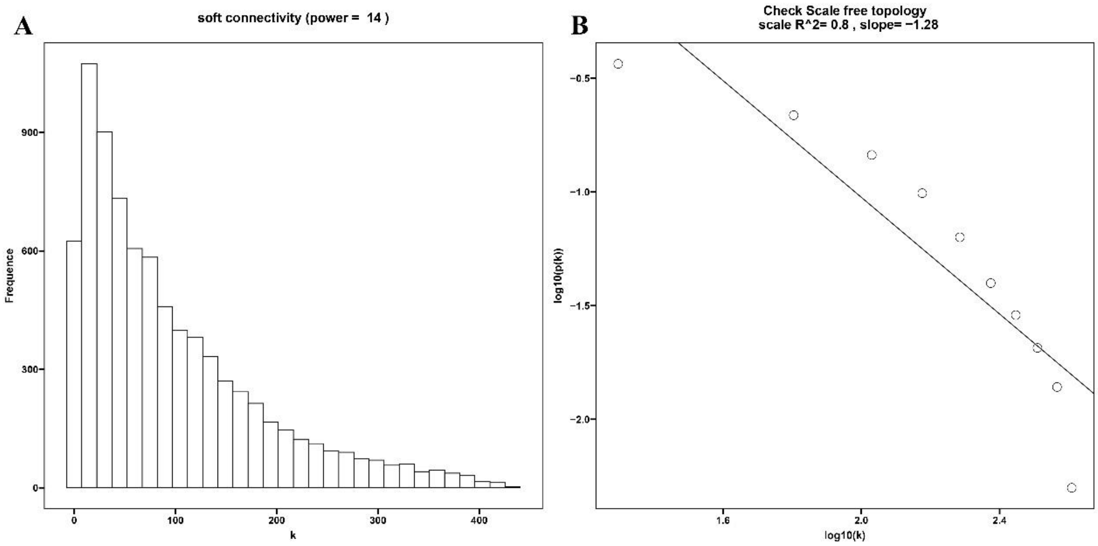

Supplementary Figure 5. Scale-free topology assessment for weighted gene co-expression network analysis.

(A) Distribution of soft connectivity at the selected power value of 14. The histogram shows the frequency distribution of connectivity values (k) across all genes included in the network construction.

(B) Verification of scale-free topology fit. The plot demonstrates the relationship between log10(k) and log10(P(k)), where k represents connectivity and P(k) represents the probability of connectivity. The scale-free topology fit index $R^2 = 0.8$ and slope = -1.28 confirm the suitability of the chosen power value for constructing biologically meaningful co-expression networks.
