# Supplementary material for: An integrated transcriptomic and metabolomic atlas reveals the temporal regulation of benzylisoquinoline alkaloid biosynthesis and transport in developing opium poppy capsules
Source: Front Plant Sci. 2026 Feb 4;17:1754793. doi: 10.3389/fpls.2026.1754793 (PMC12913367; doi:10.3389/fpls.2026.1754793)
Supplement: Supplementary file 6 [file DataSheet6.pdf]

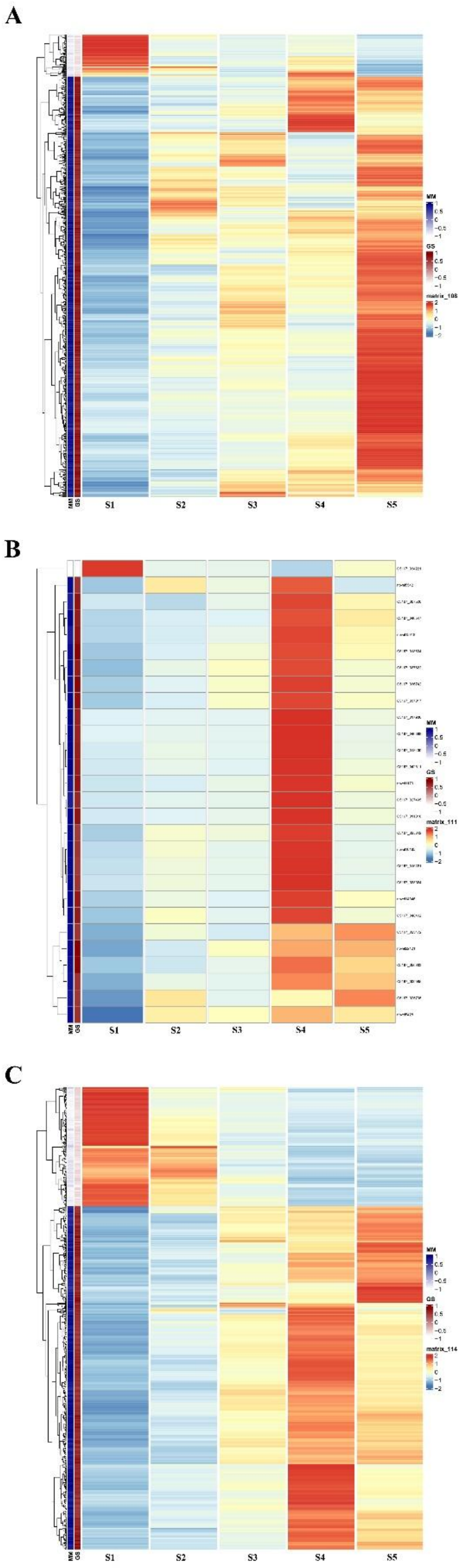

Supplementary Figure 6. Expression patterns and topological features of key co-expression modules associated with BIA accumulation.

(A) Heatmap displaying the expression profiles of hub genes in the 'brown' module (834

genes) across developmental stages S1-S5. The heatmap colors (red to blue) represent expression levels (high to low). The left sidebar shows module membership (MM) values in blue (indicating connectivity within the module) and gene significance (GS) values in red (reflecting correlation with BIA traits).

(B) Expression heatmap of hub genes in the 'steelblue' module (28 genes), showing a sharp peak at S4. The color scheme and sidebar annotations follow the same convention as in (A).

(C) Expression heatmap of hub genes in the 'blue' module, which contains homologs of the known BIA transporter BUP1. The S4-peaking pattern is consistent with the 'steelblue' module, suggesting coordinated regulation of transport-related genes during this critical developmental stage.
